# Beta-variant recombinant booster vaccine elicits broad cross-reactive neutralization of SARS-CoV-2 including Omicron variants

**DOI:** 10.1016/j.heliyon.2024.e27033

**Published:** 2024-02-28

**Authors:** Delphine Planas, Lin Peng, Lingyi Zheng, Florence Guivel-Benhassine, Isabelle Staropoli, Françoise Porrot, Timothée Bruel, Jinal N. Bhiman, Matthew Bonaparte, Stephen Savarino, Guy de Bruyn, Roman M. Chicz, Penny L. Moore, Olivier Schwartz, Saranya Sridhar

**Affiliations:** a Institut Pasteur, Paris, France; b Clinical Sciences and Operations, Sanofi, Chengdu, China; c Sanofi, Swiftwater, PA, USA; d MRC Antibody Immunity Research Unit, School of Pathology, University of the Witwatersrand, Johannesburg, South Africa; e National Institute for Communicable Diseases of the National Health Laboratory Services, Johannesburg, South Africa; f Sanofi, Waltham, MA, USA; g Centre for the AIDS Programme of Research in South Africa, University of Kwazulu-Natal, Durban, South Africa; h Sanofi, Reading, UK

**Keywords:** Vaccines, SARS CoV-2, COVID-19, Booster, Neutralizing antibody, Randomized controlled trial

## Abstract

**Background:**

SARS-CoV-2 Omicron lineage contains variants with multiple sequence mutations relative to the ancestral strain particularly in the viral spike gene. These mutations are associated inter alia with loss of neutralization sensitivity to sera generated by immunization with vaccines targeting ancestral strains or prior infection with circulating (non-Omicron) variants. Here we present a comparison of vaccine formulation elicited cross neutralization responses using two different assay readouts from a subpopulation of a Phase II/III clinical trial.

**Methods:**

Human sera from a Phase II/III trial (NCT04762680) was collected and evaluated for neutralizing responses to SARS-CoV-2 spike antigen protein vaccines formulated with AS03 adjuvant, following a primary series of two-doses of ancestral strain vaccine in individuals who were previously unvaccinated or as an ancestral or variant strain booster vaccine among individuals previously vaccinated with the mRNA BNT162b2 vaccine.

**Results:**

We report that a neutralizing response to Omicron BA.1 is induced by the two-dose primary series in 89% of SARS-CoV-2-seronegative individuals. A booster dose of each vaccine formulation raises neutralizing antibody titers that effectively neutralizes Omicron BA.1 and BA.4/5 variants. Responses are highest after the monovalent Beta variant booster and similar in magnitude to human convalescent plasma titers.

**Conclusion:**

The findings of this study suggest the possibility to generate greater breadth of cross-neutralization to more recently emerging viral variants through use of a diverged spike vaccine in the form of a Beta variant booster vaccine.

## 1 Introduction

The advent of SARS-CoV-2 Omicron sub lineages has focused attention on the need for improvement on current vaccination strategies [1]. The original COVID-19 vaccines developed with the Spike antigen from the prototype Wuhan-1 strain provide short-term protection against infection [2,3], but little is known about elicitation of cross-neutralizing antibodies and consequent protection against Omicron with variant-based booster vaccines. Here, we measured cross-reactive serum-neutralizing responses to Delta, the variants of Omicron, including BA.1, BA.2, and BA.4/5 in participants from a clinical trial (NCT04762680) [4,5] assessing the primary series and booster of different recombinant pre-fusion stabilized, transmembrane domain-deleted Spike antigen-based (CoV2 preS dTM) vaccine candidates formulated incorporating the AS03-adjuvant with the live virus S-Fuse neutralization and lentivirus based pseudoneutralization assays [[6], [7], [8], [9], [10]].

## 2 Materials and methods

### 2.1 Study population

Neutralizing antibodies generated by the primary series of the monovalent (MV) D614 vaccine, CoV2 preS dTM protein from the prototype Wuhan-1 (D614) strain, were assessed in participants from a Phase II dose-finding cohort [4]. The MV D614 primary series consisted of two injections separated by a 21-day interval, for which efficacy has been reported from a phase 3 placebo-controlled efficacy trial [11]. Sera were sampled 14 days after the second injection (D36). Samples were randomly selected from a Phase II/III clinical trial (NCT04762680) based on 4 groupings as follows in order to select the first available samples with volume and consent for additional testing: 20 seronegative subjects (negative anti-S, anti-N antibodies and RT-PCR test at enrollment) each who received either 5 μg or 10 μg dose in the adult (18–59) and elderly group (>60) and 10 seropositive subjects (prior infection with SARS-CoV-2) each who were administered 5 μg dose in the adult (18–59) and elderly group (>60).

In addition, samples from Pfizer-primed participants who received the monovalent D614 booster or a variant-containing booster in the Booster Cohorts extension of the same study were evaluated at baseline (D1) or fourteen days after the booster (D15).

Sera were evaluated against D614G, Beta, Delta variants in all participants and against BA.1 variant in all participants except older seronegative adults (Table 1).

**Table 1:** Number of participants and description of treatment groups.

	PRIMARY VACCINATION				HETEROLOGOUS BOOSTING AFTER PFIZER-PRIMING					
VAT02 Cohort	Original Cohort				Booster Cohort 1		Booster Cohort 2			
Schedule	MV(D614) prime				MV(D614) boost		MV(Beta) boost		Bivalent boost	
Antigen dose, μg	10	10	5	5	5	5	5	5	5	5
Age—yrs	18–59	≥60	18–59	≥60	18–55	≥56	18–55	≥56	18–55	≥56
Baseline serostatus	Negative	Negative	Positive	Positive	NA	NA	NA	NA	NA	NA
Timepoint(s)	D36	D36	D1, D36	D1, D36	D1, D15	D1, D15	D1, D15	D1, D15	D1, D15	D1, D15
Number of participants	20	20	10	10	20	10	20	10	20	10
Variants tested	D614G, Beta, Delta, Omicron BA.1	D614G, Beta, Delta,	D614G, Beta, Delta, Omicron BA.1		Delta, Omicron BA.1, Omicron BA.2					

NA, not applicable.

### 2.2 Human subjects for convalescent plasma

Participants from South Africa who were infected during the fourth wave of COVID-19 infection were recruited between November 25, 2021 to December 20, 2021 with n = 7 between 18 and 55 years and n = 11 greater than or equal to 56 years, and those infected during the fifth wave were enrolled between 8 and May 30, 2022 with n = 9 between 18 and 55 years and n = 4 greater than or equal to 56 years. Ethics approval was acquired for each cohort from Human Research Ethics Committees at the University of Pretoria (247/2020) and University of Cape Town (R021/2020).

### 2.3 Virus strains

The reference strain D614G (hCoV-19/France/GE1973/2020) was provided by the National Reference Centre for Respiratory Viruses hosted by Institut Pasteur (Paris, France) under the direction of Professor. S. van der Werf. This viral strain was procured via European Virus Archive goes Global (Evag) platform, which has been supported by funding from the European Union's Horizon 2020 research and innovation program under grant agreement # 653316. The Beta (B.1.351) variant (CNR 202100078) was first identified in an individual residing in Créteil, France [6]. The Delta (B.1.617.2) variant was first detected in a hospitalized patient who had returned from India. The sample was collected and sequenced by the Virology laboratory at Hopital Européen Georges Pompidou (Assistance Publique – Hopitaux de Paris) [7]. The Omicron variants BA.1 and BA.2 were provided and sequenced by the NRC UZ/KU Leuven in Leuven, Belgium [8,9]. Informed consent was provided by all patients or legal representatives for the utilization of biological materials. Variant strains, isolated from nasal swabs with Vero E6 cells underwent amplification through two or three passages. Viral stocks were titrated on Vero E6 cells using a limiting dilution technique to calculate the 50% tissue culture infectious dose, or alternatively, on S-Fuse cells. Sequencing of viruses was conducted directly on nasal swabs and after passages on Vero cells. The sequences were deposited on GISAID immediately following their generation (D614G: EPI_ISL_414631; Beta: [CNR 202100078]; Delta ID: EPI_ISL_2029113; Omicron BA.1 ID: EPI_ISL_6794907. Omicron BA.2 ID: EPI_ISL_10654979).

### 2.4 S-fuse neutralization assay

U2OS-ACE2 GFP1–10 or GFP 11 cells, also termed S-Fuse cells, were used to test the sensitivity of variants to sera of vaccines. These cells exhibit GFP fluorescence upon productive infection with SARS-CoV-2 [6]. Cells were combined at a 1:1 ratio and seeded at a density of 8 × 10^3^ cells per well in μClear 96-well plates (Greiner Bio-One). SARS-CoV-2 strains were incubated with sera at the pre-specified concentrations for 15 min at room temperature before being added to S-Fuse cells. After 18 h, cells were treated with 2% paraformaldehyde followed by washing and staining with Hoechst (1:1000 dilution; Invitrogen). Subsequently, images were captured with an Opera Phenix high-content confocal microscope from PerkinElmer. Syncytia and nuclei counts were quantified utilizing Harmony software (PerkinElmer). The percentage of neutralization was determined using syncytia count and the following formula: 100 x (1 – [value with serum – value in ‘noninfected’]/[value in ‘no serum’ − value in ‘noninfected’]). The neutralizing activity of each serum was quantified as the half maximal effective dilution (ED50), computed through a reconstructed neutralization curve at varying concentrations. Prior to use, the sera underwent heat-inactivated for 30 min at 56 °C.

### 2.5 Pseudovirus neutralization assay

Methods have been previously described elsewhere (Bhiman J et al.) [10]. In brief, MF cells overexpressing human ACE2 (293T/ACE2) were generously provided by M. Farzan (Scripps Research). These cells were cultured in DMEM (Gibco BRL Life Technologies) supplemented with 10% heat-inactivated fetal bovine serum (FBS) and 3 μgml^−1^ puromycin at standard conditions (37 °C, 5% CO_2_). Upon reaching confluency, cell monolayers were dissociated using 0.25% trypsin in 1 mM EDTA solution (Gibco BRL Life Technologies). The SARS-CoV-2, Wuhan-1 spike protein, cloned into pCDNA3.1 underwent mutagenesis utilizing QuikChange Lightning Site-Directed Mutagenesis kit (Agilent Technologies) to include specific mutations such as D614G (ancestral D164G) or L18F,D80A, D215G, Δ242–244, K417 N, E484K, N501Y, D614G, A701V (Beta) or Δ69–70, T915I, Δ143-145, Δ211, L212I, ins 214 EPE, G339D, S371L, S373P, S375F, K417 N, N440K, G446S, S477 N, T478K, Q493R, G496S, Q498R, N501Y, Y505H, T547K, D614G, N679K, P681H, N764K, D796Y, N856K, Q954H, N969K, L981F (Omicron BA.1) or T19I, L24S, Δ25-27, Δ69-70, G142D, V213G, G339D, S371F, S373P, S375F, T376A, D405 N, R408S, K417 N, N440K, L452R, S477 N, T478K, E484A, F486V, Q498R, N501Y, Y505H, D614G, H655Y, N679K, P681H, N764K, D796Y, Q954H, N969K (Omicron BA.4/BA.5). Pseudoviruses were generated through co-transfection with a lentiviral backbone (HIV-1 pNL4.luc encoding the firefly luciferase gene) and respective SARS-CoV-2 spike plasmids using PEIMAX (Polysciences). Culture supernatants were clarified by filtration (0.45 μM filter) and stored at −80 °C. Plasma samples were heat-inactivated and clarified by centrifugation before being serially diluted and incubated with pseudovirus at standard conditions. After 1 h, cells incubated for 72 h were added at 1 × 10^4^ cells per well, and luminescence was measured using PerkinElmer Life Sciences Model Victor X luminometer. Neutralization was determined by the reduction in luciferase gene expression following single-round infection of 293T/ACE2.MF cells with spike-pseudo typed viruses. Titers were calculated as the reciprocal plasma dilution (ID_50_) or monoclonal antibody concentration (IC_50_) resulting in a 50% reduction in relative light units. The equivalency of assays was confirmed through participation in the SARS-CoV-2 Neutralizing Assay Concordance Survey Concordance Survey 1 conducted by EQAPOL and VQU, Duke Human Vaccine Institute. Cell-based neutralization assays using live virus or pseudovirus have consistently shown high concordance, with highly correlated 50% neutralization titers (Pearson r = 0.81–0.89).

## 3 Results

### 3.1 Characteristics of the study profile

Table 1 summarizes the study participants and treatment groups: age group, sero status, number of participants, dosing, vaccine cohort, treatment schedule, and variant immunogenicity testing.

### 3.2 Primary vaccine immunogenicity

**Fig. 1:**
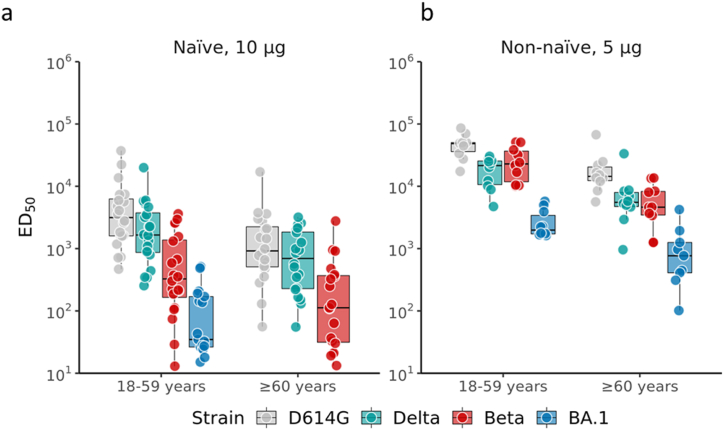
**Neutralizing antibody responses to D614G, Beta, Delta and BA.1 after primary vaccination using AS03-adjuvanted recombinant-protein pre-S.dTM with prototype D614 protein** Live virus neutralization assay antibody titers against the wild-type D614G, Delta, Beta and BA.1 viruses measured in sera collected 14 days after a two-injection primary series of the MV D614 vaccine in naïve (**panel a**) and non-naïve (**panel b**) individuals 18–59 years of age and 60 years and above. The 50% inhibitory dilution (ID50) neutralizing antibody titers are presented here. Each circle represents individual participants. The center line of the boxplot indicates the median, and the lower and upper edges of the boxplot indicate the first quartile(Q1) and third quartile(Q3). The lower and upper whiskers that extend from the IQR represent Q1-1.5 × IQR and Q1+1.5 × IQR, respectively. Points that beyond the whiskers indicate potential outliers in each sub-group. The individual data points are jittered horizontally and overlaid over Boxplot for better visualization. The lower limit of detection of the assay was 10. Values below the lower limit of detection were assigned a value of 5.

In naïve 18–59 yo participants, high neutralizing responses to D614G and Delta (Geometric Mean Titers [GMTs]: 3294 and 1680, respectively) were observed (Table 2) with titers to Delta 2-fold lower compared to D614G strain (Fig. 1). In seropositive participants, we observed high neutralizing antibody titers (GMTs>1000) across D614G, Delta, and Beta variants (Fig. 1). These responses were within the range of those previously measured with the same assay in individuals vaccinated with BNT162b2 and ChAdOx1 vaccines [6,7] and provide an immunological basis for the clinical efficacy against the Delta variant observed in the aforementioned phase 3 trial [11].

**Table 2:** Summary of neutralizing antibody against SARS-CoV-2 strains 14 days after the second injection and fold change compared to D614G strain – phase 2 dose-finding cohort.

Age group	Group	D614G			Beta			Delta			BA.1			D614G/Beta		D614G/Delta		D614G/BA.1	
		M	GMT	95% CI	M	GMT	95% CI	M	GMT	95% CI	M	GMT	95% CI	Fold change	95% CI	Fold change	95% CI	Fold change	95% CI
18–59 years	Negative: 10 μg	20	3294	(1922; 5644)	20	361	(174.32; 746.33)	20	1680	(985; 2864)	19	51	(27; 95)	9.1	(6.4; 13.0)	2.0	(1.6; 2.5)	61.7	(46.9; 81.2)
	Positive: 5 μg	10	43729	(31526; 60655)	10	22322	(14129.87; 35263.93)	10	16255	(10463; 25253)	10	2460	(1736; 3487)	2.0	(1.2; 3.2)	2.7	(2.1; 3.5)	17.8	(11.2; 28.2)
≥ 60 years	Negative: 10 μg	20	993	(542; 1816)	20	107	(49; 231)	20	611	(352; 1058)	–	–	–	9.3	(5.9; 14.6)	1.6	(1.2; 2.2)	–	–
	Positive: 5 μg	10	15816	(9823 25465)	10	4686	(2549; 8614)	10	5655	(2987; 10704)	9	706	(304; 1640)	3.4	(2.3; 5.0)	2.8	(2.1; 3.7)	22.8	(10.4; 49.7)

GMT: geometric mean titer.

Fold change: geometric mean of individual titer against D614G/other strains.

In contrast with a previous report where no detectable cross-reactive anti-BA.1 antibodies were observed in most individuals after a primary series of BNT162b2 and ChAdOx1 vaccines [6], neutralizing antibodies to Omicron BA.1 were observed in 17 of 19 younger seronegative adults after MV D614 recombinant-protein primary series.

In younger seropositive participants, anti-BA.1 cross-reactive antibodies were >40-fold higher (GMTs:2460) compared to seronegative participants (GMTs:51). The decrease in anti-BA.1 titers relative to the D614G strain was also lower in seropositive (18-fold) than in seronegative participants (62-fold) (Table 2). This observation suggests that administering a booster dose of the CoV2 preS dTM recombinant-protein vaccine may induce significant cross-reactivity to Omicron.

### 3.3 Booster vaccine immunogenicity

We next assessed neutralizing antibodies to Delta, Omicron BA.1, BA.2, and BA.4/5 in individuals from the Phase III booster cohorts [5]. Three formulations of the CoV2 preS dTM booster were each administered 4–10 months following primary vaccination with mRNA (Pfizer/BioNTech and Moderna) or adenoviral-vectored (AstraZeneca and Janssen) vaccines. Monovalent formulations contained CoV2 preS dTM protein derived from the prototype Wuhan-1 strain or Beta variant (5 μg of MV[D614] or MV[Beta], respectively) and the bivalent (BV) formulation included 2.5 μg each of D614 and Beta preS dTM protein with each formulation combined with AS03 adjuvant. Participants receiving MV(D614) were enrolled in July and August 2021 and those receiving either MV(Beta) or BV were enrolled in November and December 2021. Sera were collected 15 days after the booster administration (D15). For each formulation, 20 and 10 participants 18–55 yo and ≥56 yo, respectively, who had been previously administered with two doses of mRNA BNT162b2 were randomly selected in order to select the first available samples with volume and consent for additional testing (negative anti-N antibodies and RT-PCR test at enrollment). These sera were evaluated against Delta, BA.1 and BA.2 variants in the S fuse microneutralization assay.

**Fig. 2:**
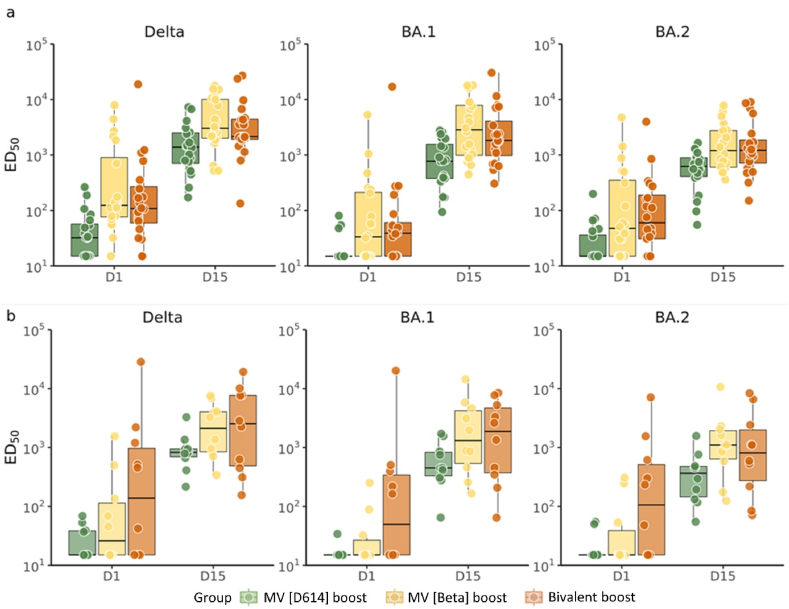
**Neutralizing antibody responses to Delta, BA.1 and BA.2 after boosting with CoV2 preS dTM recombinant protein with AS03 adjuvant from prototype and variant vaccines.** Live virus neutralization assay titers against Delta, BA.1 and BA.2 in sera collected prior (D1) and 15 days (D15) after booster vaccination of 18–55-year-old (**Panel a**) and ≥56-year-old (**Panel b**) individuals having previously received two doses of the BNT162b2 vaccine. The booster formulations were a monovalent D614 spike (MV[D614], 5 μg), a monovalent Beta (MV[Beta], 5 μg) and bivalent (a mix of MV[D614] and MV[Beta], 5 μg each). The 50% inhibitory dilution (ID50) neutralizing antibody titers are presented here. Each circle represents individual participants. The center line of the boxplot indicates the median, and the lower and upper edges of the boxplot indicate the first (Q1) and third quartiles (Q3). The lower and upper whiskers that extend from the IQR represent Q1-1.5 × IQR and Q1+1.5 × IQR, respectively. Points beyond the whiskers indicate potential outliers. The individual data points are jittered horizontally and overlaid over Boxplot for better visualization. The lower limit of detection of the assay was 10. Values below the lower limit of detection were assigned a value of 5.

**Fig. 3:**
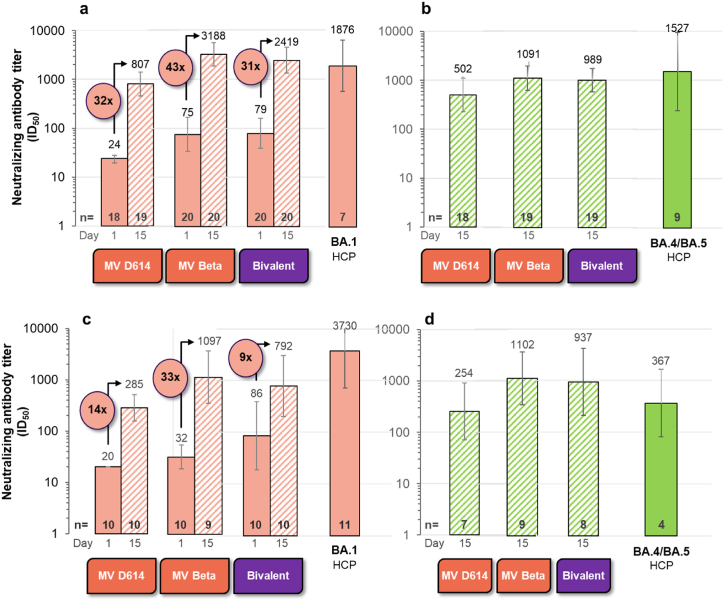
**Neutralizing antibody responses to Omicron BA.1 and BA.4/5 after boosting with CoV2 preS dTM recombinant protein with AS03 adjuvant from prototype and variant vaccines.** Pseudovirus neutralization assay titers against Omicron BA.1 (**Panels a** and **c**) and BA.4/5 (**Panels b** and **d**) in sera collected prior (D1) and/or 15 days (D15) after booster vaccination of 18–55-year-old (**Panels a** and **b**) and ≥56-year-old (**Panels c** and **d**) individuals having previously received two doses of the BNT162b2 vaccine. The booster formulations were a monovalent D614 spike (MV[D614], 5 μg), a monovalent Beta (MV[Beta], 5 μg) and bivalent (a mix of MV[D614] and MV[Beta], 2.5 μg each). Human convalescent plasma (HCP) results from Penny Moore Lab, unpublished data.

The three booster vaccines induced significant increases in neutralizing titers to the three variants in younger and older adults, when compared to pre-boost levels (D1) (Fig. 2). When compared to Delta, neutralizing antibody titers to BA.1 and BA.2 were 1–2 fold and ∼2–3 fold lower, respectively (Table 2). This relative decrease in titers to BA.1 is much lower in the MV(Beta) and BiV groups than after primary MV D614 vaccination. Comparison of the three booster formulations needs to be undertaken with caution given the small subset of participants and differences in baseline antibody levels due to prior vaccination or infection. These limitations notwithstanding, we observed higher cross-neutralizing titers to Delta, BA.1 and BA.2 with MV(Beta) or BV than with MV(D614) boosting (Fig. 2) in older and younger adults. The titers induced by the MV(Beta) booster were higher than those observed after the primary series of MV D614 vaccine. The same samples were additionally examined for cross neutralization efficacy against Omicron BA.4/5 in a pseudovirus neutralization assay using D614G, Omicron BA.1, and convalescent patient sera as reference standards. When compared to D614G, neutralizing antibody titers to BA.1 and BA.4/5 were 3–5 fold and ∼7–8 fold lower, respectively (Table 3). This relative decrease in titers is much lower in the MV(Beta) and BiV groups than that observed after primary MV D614 vaccination. The observed higher cross-neutralizing titers to D614G, BA.1 and BA.4/5 with MV(Beta) or BV than with MV(D614) boosting (Fig. 3) in older and younger adults is consistent with that observed using the S fuse microneutralization assay. The titers induced by the MV(Beta) booster were higher than those observed after the primary series of MV D614 vaccine.

**Table 3:** Summary of neutralizing antibody against SARS-CoV-2 strains and fold change compared to Delta strain – phase 3 booster cohorts.

Age group	Formulation	Time point/Ratio	Delta			BA.1			BA.2			Delta/BA.1		Delta/BA.2	
			M	GMT/GMFR	95% CI	M	GMT/GMFR	95% CI	M	GMT/GMFR	95% CI	Fold change	95% CI	Fold change	95% CI
18–55 years	MV[D614] boost	D1	20	34	(22; 52)	20	18	(15; 235)	20	23	(16; 33)	1.8	(1.3; 2.6)	1.5	(1.1; 2.0)
		D15	20	1281	(800; 2053)	20	685	(4315; 1089)	20	508	(334; 775)	1.9	(1.5; 2.4)	2.5	(1.8; 3.6)
		D15/D1	20	38	(22; 64)	20	37	(25; 55)	20	22	(14; 35)				
	MV[Beta] boost	D1	20	226	(99; 516)	20	60	(27; 134)	20	82	(36; 187)	3.8	(2.6; 5.4)	2.8	(2.1; 3.7)
		D15	20	3508	(2090; 5889)	20	2874	(1643; 5026)	20	1356	(878; 2095)	1.2	(0.8; 1.8)	2.6	(1.8; 3.7)
		D15/D1	20	16	(7; 37)	20	48	(18; 130)	20	17	(7; 41)				
	Bivalent boost	D1	20	148	(67; 328)	20	52	(23; 113)	20	84	(42; 170)	2.9	(2.0; 4.1)	1.8	(1.2; 2.5)
		D15	20	2838	(1649; 4885)	20	2141	(1246; 3679)	20	1321	(793; 2199)	1.3	(1.0; 1.8)	2.2	(1.5; 3.1)
		D15/D1	20	19	(9; 43)	20	42	(20; 85)	20	16	(7; 36)				
≥ 56 years	MV[D614] boost	D1	10	24	(15; 37)	10	16	(14; 120)	10	19	(13; 28)	1.5	(1.0; 2.1)	1.2	(0.7; 2.2)
		D15	10	812	(489; 1349)	10	489	(248; 964)	10	300	(148; 609)	1.7	(1.2; 2.4)	2.7	(1.8; 4.1)
		D15/D1	10	34	(19; 62)	10	30	(15; 61)	10	16	(7; 35)				
	MV[Beta] boost	D1	10	55	(17; 182)	10	26	(13; 52)	10	30	(13; 73)	2.1	(1.1; 4.1)	1.8	(1.0; 3.2)
		D15	10	1926	(899; 4125)	10	1427	(507; 4012)	10	1006	(402; 2518)	1.4	(0.89; 2.06)	1.9	(1.0; 3.7)
		D15/D1	10	35	(7; 168)	10	56	(14; 229)	10	33	(10; 106)				
	Bivalent boost	D1	10	181	(27; 1222)	10	101	(18; 559)	10	128	(26; 636)	1.8	(1.2; 2.7)	1.4	(0.8; 2.6)
		D15	10	1994	(605; 6573)	10	1237	(373; 410)	10	763	(236; 2467)	1.6	(1.3; 2.0)	2.6	(1.7; 4.0)
		D15/D1	10	11	(4; 30)	10	12	(3.86; 38.95)	10	6	(3; 13)				

GMT: geometric mean titer; GMFR: geometric mean fold rise (individual titer at D15/D1).

Fold-change: geometric mean of individual titer against D614G/other strains.

Human convalescent plasma (HCP) collected from hospitalized individuals with mild to moderate COVID-19 were tested against BA.1 [12] and BA.4/5 [13] to contextualise the vaccine elicited titers (Fig. 3). In all individuals, regardless of age, both the MV (Beta) and BV boosters elicited similar (1.3–1.5-fold) or higher titers (3–4.7-fold) against BA.1 and BA.4/5, compared to individuals infected with these variants (HCP). In contrast, individuals boosted with the MV (D614), had lower titers to BA.1 and BA.4/5 compared to HCP titers.

## 4 Discussion

Taken together, our findings strongly suggest that a Beta variant-spike-based recombinant booster vaccine represents a valuable option in a situation of Omicron predominance and continuous SARS-CoV-2 evolution. Across two independent laboratories applying distinct laboratory methods for evaluation of antibodies with neutralizing activity against SARS- CoV-2, we found that a monovalent Beta variant spike protein booster vaccine with AS03 adjuvant yielded the highest neutralizing antibody responses.

The improved magnitude of neutralizing titers after the Beta variant booster noted in the current study are consistent with the observations from an independently conducted trial [14]. The enhanced neutralizing response to a monovalent heterologous (Beta strain) booster vaccine is all the more intriguing, given that a primary series of the bivalent vaccine showed clinical efficacy for protection against symptomatic COVID-19 in a pandemic period marked by the circulation of Delta and Omicron BA.1 [15]. The bivalent booster showed no meaningful advantage in terms of neutralizing antibody magnitude over the monovalent Beta booster vaccine consistent with a recently published 2nd booster study [16]. Our data suggests that within the framework of hybrid immunity, continual boosting with D614 immunogens, results in a plateauing of the antibody response, while exposure to Beta immunogens, which are move divergent, resulted in the expansion of cross-reactivity, likely because of the affinity maturation of D614-primed B cells. This was confirmed after the UK Spring COVID booster campaign where the recombinant Beta variant booster demonstrated similar effectiveness as the mRNA BA.4/5 + ancestral bivalent booster targeting the circulating Omicron XBB.1.5 variant of concern [17].

The present study evaluated the major immune measure thus far shown to predict vaccine efficacy [[18], [19], [20]], namely the presence and magnitude of neutralizing antibodies to the ancestral strain. Markers to cellular immunity are also likely to contribute to protection against infection and severe manifestations of disease but were not assessed here. Prior work [21] has shown two doses of the ancestral strain primary series vaccine generated a high magnitude of polyfunctional, Th1-oriented CD4^+^ T cells. Ongoing studies will further evaluate the cellular component of the immune response to booster doses of these vaccine formulations.

One limitation of the present study is the sample size of the random subset selected for testing. In that respect, the findings are consistent with the overall study results, as evaluated based on neutralizing results from a third pseudovirus neutralization assay. Differences in the period of collection of samples for Booster Cohort 1 and Booster Cohort 2 were accompanied by differences in rates of pre-booster prevalence of prior infection among previously vaccinated participants. Given the differences in the magnitude of the neutralizing responses noted for the booster vaccine formulations, it is unlikely that the minor difference in frequency of prior exposure would account for the differences observed. Despite these limitations, our results substantiate the update of future COVID-19 vaccines with immunogens that are more divergent than dominant spike variants and which may circulate at relatively low frequencies.

## Ethical statement

Study has been approved from Institutional Ethical Committee and the clinical trial identifier is NCT04762680.

## Data availability

Qualified researchers can request access to patient-level data and related study documents, including the clinical study report, study protocol with any amendments, blank case report forms, statistical analysis plan, and dataset specifications. Patient-level data will be anonymized, and study documents will be redacted to protect the privacy of trial participants. Further details on Sanofi's data sharing criteria, eligible studies, and process for requesting access can be found at https://vivli.org/.

## CRediT authorship contribution statement

**Delphine Planas:** Writing – review & editing, Methodology, Investigation. **Lin Peng:** Writing – review & editing, Visualization, Formal analysis. **Lingyi Zheng:** Writing – review & editing, Visualization, Formal analysis. **Florence Guivel-Benhassine:** Writing – review & editing, Methodology, Investigation. **Isabelle Staropoli:** Writing – review & editing, Methodology, Investigation. **Françoise Porrot:** Writing – review & editing, Methodology, Investigation. **Timothée Bruel:** Writing – review & editing, Methodology, Investigation. **Jinal N. Bhiman:** Writing – review & editing, Methodology, Investigation. **Matthew Bonaparte:** Writing – review & editing, Project administration, Data curation. **Stephen Savarino:** Writing – review & editing, Supervision, Conceptualization. **Guy de Bruyn:** Writing – review & editing, Writing – original draft, Project administration, Investigation, Funding acquisition. **Roman M. Chicz:** Writing – review & editing, Supervision, Resources, Project administration, Funding acquisition, Conceptualization. **Penny L. Moore:** Writing – review & editing, Supervision, Resources, Methodology. **Olivier Schwartz:** Writing – review & editing, Supervision, Resources, Methodology. **Saranya Sridhar:** Writing – review & editing, Writing – original draft, Supervision, Funding acquisition, Conceptualization.

## Declaration of competing interest

The authors declare that they have no known competing financial interests or personal relationships that could have appeared to influence the work reported in this paper.

Lingyi Zheng reports a relationship with Sanofi, Swiftwater, PA, USA that includes: employment. Stephen Savarino reports a relationship with Sanofi, Swiftwater, PA, USA that includes: employment. Saranya Sridhar has patent pending to Assignee. Roman M Chicz has patent pending to Assignee. Guy de Bruyn has patent pending to Asignee. Stephen Savarino has patent pending to Assignee. If there are other authors, they declare that they have no known competing financial interests or personal relationships that could have appeared to influence the work reported in this paper.
